# Linear regression analysis for complete blood count parameters during radiotherapy

**DOI:** 10.1007/s00066-024-02344-1

**Published:** 2025-01-10

**Authors:** Aniwat Berpan, Nattapatch Janhom

**Affiliations:** 1 Department of Radiology, Faculty of Medicine Vajira Hospital, Navamindradhiraj University, 681 Samsen Road, Dusit, 10300 Bangkok, Thailand; 2 Department of Radiology, Samut Sakhon Hospital, 74000 Samut Sakhon, Thailand

**Keywords:** Anemia, Cumulative dose, Hemoglobin, Predictive model, Radiotherapy

## Abstract

**Objective:**

This study aimed to evaluate the correlations between complete blood count (CBC) during radiotherapy and patient and treatment factors.

**Patients and methods:**

Data of cancer patients, including age, sex, concurrent chemotherapy (CCRT), radiotherapy dose (equivalent dose in 2‑Gy fractions with an alpha/beta value of 10 Gy, EQD2Gy10), radiotherapy location, and baseline CBC were collected. Linear regression was used to determine results during radiation. Validation data comprised 20% of the whole cohort.

**Results:**

A total of 496 radiotherapy courses and 1884 weekly CBC results during treatment were analyzed. Baseline hemoglobin (Hb) was positively associated with subsequent Hb. Each 1 g/dL increase in baseline Hb predicted a 0.73 g/dL increase in Hb during treatment (95% confidence interval [CI] 0.7–0.76). Male sex was associated with a 0.16 g/dL higher Hb (95% CI 0.04–0.29), while female sex showed the opposite trend. CCRT was associated with a 0.18 g/dL reduction in Hb (95% CI −0.33 to −0.03). Radiotherapy to the pelvis, bone, and head and neck regions resulted in Hb reductions of 0.18, 0.34, and 0.94 g/dL, respectively (95% CI −0.33 to −0.03, −0.53 to −0.15, and −1.26 to −0.62, respectively), while brain irradiation increased Hb by 0.22 g/dL (95% CI 0.05–0.38). Age, cumulative dose, and thoracic irradiation did not show a significant correlation with Hb changes. Adjusted R‑squared for the development and validation data were 0.6 and 0.71 for Hb, 0.42 and 0.11 for white blood cell count, 0.36 and 0.32 for neutrophils, 0.42 and 0.06 for absolute neutrophil count, and 0.43 and 0.36 for platelets, respectively.

**Conclusion:**

Hb levels during radiotherapy could be explained using linear regression, although they did not negatively correlate with cumulative dose.

**Supplementary Information:**

The online version of this article (10.1007/s00066-024-02344-1) contains supplementary material, which is available to authorized users.

## Introduction

Bone marrow, which has a significant role in the production of blood cells, is distributed throughout bones and easily unintentionally involved in radiation fields. An abnormal complete blood count (CBC) is common in cancer patients, not only due to prior comorbidities or cancer itself but also due to treatments for the cancer, including both systemic and radiation therapies. Anemia is correlated with poor outcomes in cancer patients undergoing radiotherapy [1, 2], while leukopenia is a risk factor for infection, especially in patients with neutropenia. Furthermore, thrombocytopenia can lead to a bleeding tendency. These situations affect not only oncologic outcomes because of treatment interruption and delay, but also patients’ quality of life due to symptoms caused by the complications or following treatments. Apart from that, they can be reasons for an excessive treatment cost due to supportive treatments—not only blood transfusions but also medications or even hospitalization. Understanding the association between CBC parameters during radiotherapy and both patient and treatment factors could help to prevent or minimize possible subsequent morbidities, not to mention complications during radiation treatment, and even mortality. In accordance with previous knowledge, the magnitude of the blood cell concentration change depends on the dose, volume, and location of radiation [3]. Aside from these, other factors affecting bone marrow function, including systemic treatment, especially chemotherapy, and abnormal baseline CBC values, can inevitably lead to cytopenia. Besides, venipuncture for CBC monitoring during the therapy can be a burden for both patients and healthcare personnel. Predictive models for the values could help to determine whether a patient requires the procedure, which could individualize the care plan for each patient and eliminate unnecessary expenses.

## Materials and methods

Data of radiation courses administered between 1 May 2020 and 31 May 2022 were retrospectively collected from electronic health records. All courses delivered during the period were included. Courses without CBC results and complete data were excluded from the study. CBC results were randomly assigned to development or validation datasets using random sampling, with a 4:1 ratio. This study was approved by the institutional review board.

Due to the limited number of dosimetrists and medical physicists during the period, all treatments were performed using three-dimensional conformal radiotherapy (3DCRT). CBC, including hemoglobin (Hb) level, white blood cell (WBC) count, neutrophil level, absolute neutrophil count (ANC), and platelet count, was acquired 1 week prior to initiation of radiotherapy and then weekly until the end of treatment. The indication for granulocyte colony-stimulating factor (G-CSF) administration was ANC below 1500/mm^3^ in patients receiving concurrent chemoradiation (CCRT) and below 1000/mm^3^ in patients receiving radiotherapy alone. Platelet concentrate and packed red blood cells were indicated if patients had platelet levels lower than 50,000/mm^3^ and Hb levels below 8 g/dL, respectively. Chemotherapy and radiation were discontinued if patients had ANC below 1500/mm^3^ and 500/mm^3^, respectively. To calculate the cumulative dose for each CBC result, an equivalent dose in 2‑Gy fractions (EQD2) formula was used with an alpha/beta value of 10 Gy for bone marrow in terms of early organ damage [4]. The dose rate was 400 monitor units per minute.

To determine whether CBC parameters during treatment and both patient and treatment variables (including age, sex, CCRT, cumulative radiotherapy dose [EQD2Gy10], location, and baseline values) were associated, the Pearson product–moment correlation coefficient was calculated, and multiple linear regression analyses with adjustment for all aforementioned covariates were performed. Since treatment location is a known factor associated with CBC parameter change [3], this variable was included in the analyses. All tests were considered statistically significant at *p*-value ≤ 0.05. PASW Statistics (SPSS) 28.0 (IBM Corp., Armonk, NY, USA) was used for the analysis.

## Results

A total of 1345 radiation courses were assessed for eligibility, and 496 courses with complete data were included. Patients’ mean age was 56 years (standard deviation [SD] 12.3), 74% were female, and 103 courses were delivered concurrently with chemotherapy (20.8%). Most treatments were conventionally fractionated (61.7%), the rest were hypofractioned. The number of fractions was 5–35 (median 25; interquartile range 10–25), and the dose per fraction ranged from 1.8 to 4 Gy (mean 2.33 Gy; SD 0.5). An EQD2Gy10 of 45 Gy was the mean total dose (SD 10.24). Most patients underwent irradiation to the breast (39.7%), followed by the pelvis (30.2%), brain (14.5%), bone (11.1%), head and neck (2.4%), and thorax (2%). Table 1 shows the patient and radiotherapy characteristics for each course. A total of 1884 CBC results were analyzed after exclusion of those with incomplete data. Mean cumulative radiation dose was 28.39 Gy (SD 13.91). Patient and treatment characteristics for each CBC result, including the whole, development, and validation cohorts, are noted in Table 2.

**Table 1:** Patient characteristics at baseline for each course

Characteristic	*N* = 496
Age (years), mean (SD)	56 (12.3)
Female	367 (74%)
Concurrent chemoradiotherapy	103 (20.8%)
Total radiation dose (EQD2Gy10), mean (SD)	45 (10.24)
Conventional fractionation	306 (61.7%)
Hypofractionation	190 (38.3%)
*Location*	
Breast	197 (39.7%)
Pelvis	150 (30.2%)
Brain	72 (14.5%)
Bone	55 (11.1%)
Head and neck	12 (2.4%)
Thorax	10 (2%)
*Baseline, mean (SD)*	
Hb (g/dL)	11.54 (1.59)
WBC (/mm^3^)	7654 (4168.63)
Neutrophils (%)	69.91 (11.17)
ANC (/mm^3^)	5540 (3551.71)
Platelet (/mm^3^)	299,234 (105,356.32)

*ANC* absolute neutrophil count, *EQD2Gy10* equivalent dose in 2‑Gy fractions with an alpha/beta value of 10 Gy, *Hb* hemoglobin, *SD* standard deviation, *WBC* white blood cell

**Table 2:** Patient characteristics at baseline for each weekly complete blood count result

Characteristic	Whole cohort (*n* = 1884)	Development cohort (*n* = 1507)	Validation cohort (*n* = 377)
Age (years), mean (SD)	55.6 (12.2)	56.2 (12.2)	52.84 (11.64)
Female	1451 (77%)	1205 (80%)	246 (65.25%)
Concurrent chemoradiotherapy	533 (28.3%)	444 (29.5%)	89 (23.6%)
Cumulative radiation dose (EQD2Gy10), mean (SD)	28.39 (13.91)	28.31 (13.92)	28.68 (13.85)
*Location*			
Breast	814 (43.21%)	648 (43%)	166 (44.03%)
Pelvis	714 (37.9%)	593 (39.35%)	121 (32.1%)
Brain	168 (8.92%)	112 (7.43%)	56 (14.85%)
Bone	119 (6.32%)	97 (6.44%)	22 (5.84%)
Head and neck	41 (2.18%)	39 (2.59%)	2 (0.53%)
Thorax	28 (1.49%)	18 (1.19%)	10 (2.65%)
*Baseline, mean (SD)*			
Hb (g/dL)	11.59 (1.59)	11.07 (1.27)	13.68 (0.83)
WBC (/mm^3^)	7306 (3498.26)	7193 (3600.84)	7759 (3016.04)
Neutrophils (%)	68.64 (10.69)	68.31 (10.91)	69.94 (9.7)
ANC (/mm^3^)	5166 (3015.73)	5058 (3014.94)	5598 (2872.21)
Platelet (/mm^3^)	294,614 (102,331.76)	298,749 (107,874.93)	278,085 (74,095.53)

*ANC* absolute neutrophil count, *EQD2Gy10* equivalent dose in 2‑Gy fractions with an alpha/beta value of 10 Gy, *Hb* hemoglobin, *SD* standard deviation, *WBC* white blood cell

**Fig. 1:**
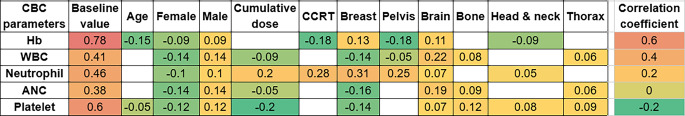
Pearson correlation between complete blood count (*CBC*) parameters during radiotherapy and patient and treatment characteristics. This heatmap demonstrates the strength of correlations between various blood parameters and patient and treatment characteristics. The color intensity indicates the correlation strength, with values closer to 1 or −1 representing stronger relationships. The blank cells represent nonsignificant values. *ANC* absolute neutrophil count, *CCRT* concurrent chemoradiation, *Hb* hemoglobin, *WBC* white blood cell

The Pearson correlation analysis showed moderate-to-strong positive relationships between baseline values and values measured during treatment for various CBC parameters. Specifically, the correlation coefficients were 0.78 for Hb level, 0.41 for WBC count, 0.46 for neutrophil level, 0.38 for ANC, and 0.6 for platelet count (all with a *p*-value < 0.001). Although most of the other correlations were statistically significant, their strengths were weak (Fig. 1 and Table S1).

**Fig. 2:**
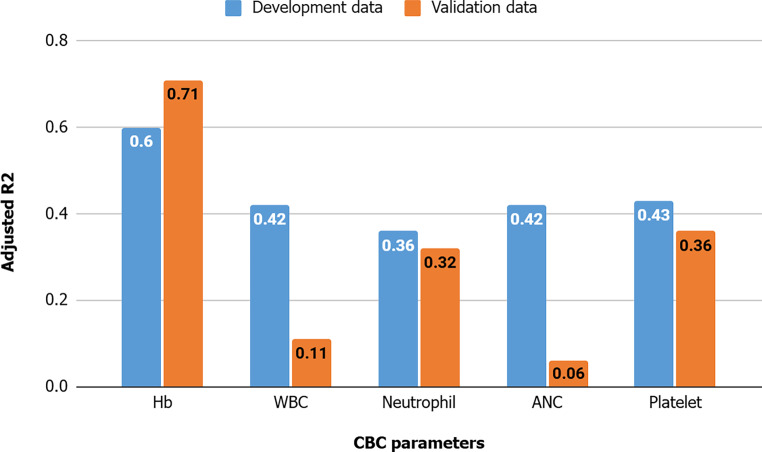
Adjusted R‑squared of linear regression analysis for each complete blood count (*CBC*) parameter during radiation treatment. *ANC* absolute neutrophil count, *Hb* hemoglobin, *WBC* white blood cell

**Fig. 3:**
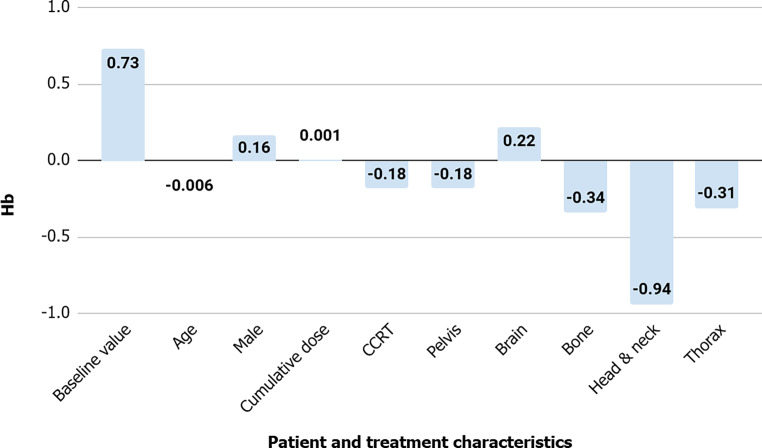
Coefficients for multiple linear regression analysis of hemoglobin during radiation treatment. *CCRT* concurrent chemoradiation, *Hb* hemoglobin

For the development and validation data, adjusted R‑squared values were 0.6 and 0.71 for Hb, 0.42 and 0.11 for WBC, 0.36 and 0.32 for neutrophils, 0.42 and 0.06 for ANC, and 0.43 and 0.36 for platelets (Fig. 2). Other performance metrics are presented in Table S2. A higher baseline Hb level was associated with a higher Hb level during radiotherapy (0.73 g/dL of subsequent Hb for each g/dL of initial Hb; 95% CI 0.7–0.76). Older age was associated with a lower Hb level (−0.006 g/dL for a year; 95% CI −0.01 to −0.002). Male sex resulted in an Hb increase of 0.16 g/dL, while female sex demonstrated the opposite direction. Additionally, lower Hb was associated with CCRT (−0.18 g/dL; 95% CI −0.33 to −0.03) and treatment to the pelvis (−0.18 g/dL; 95% CI −0.33 to −0.03), bone (−0.34 g/dL; 95% CI −0.53 to −0.15), and head and neck (0.94 g/dL; 95% CI −1.26 to −0.62). Brain irradiation was associated with a 0.22 g/dL higher Hb level (95% CI 0.05–0.38). Cumulative dose and thoracic irradiation did not affect Hb changes (95% CI −0.002 to 0.004 and −0.68 to 0.06, respectively; Fig. 3). Table S3 shows other interceptions and coefficients for multiple linear regression analyses.

## Discussion

One aim of the present study was to evaluate the impact of pretreatment values, age, sex, CCRT, cumulative radiation dose, and location of irradiation on CBC parameters during radiotherapy. Multiple linear regression analyses were performed. The model explained approximately 70% of the variance in Hb level during radiotherapy. Statistically significant variables included baseline Hb level; age; male sex; CCRT; and pelvic, brain, bone, and head and neck sites. Only cumulative radiation dose and a thoracic site were nonsignificant. During the therapy, regression analysis explained approximately 10%, 30%, 6%, and 40% of the variance in WBC count, neutrophil level, ANC, and platelet count, respectively.

In correlation analysis, despite the weak correlation between cumulative dose and CBC parameters, negative associations of WBC count, ANC, and particularly platelet count were evident. In contrast, the strength of the association of Hb level was remarkably weak. Besides, in multiple regression analysis, the coefficient of cumulative dose was not statistically significant for Hb level. These results are consistent with the results of a study by Sini and colleagues, which showed that the Hb level remained stable throughout postoperative whole-pelvis intensity-modulated radiotherapy (IMRT) in prostate cancer patients, and thereafter for at least 1 year, while WBC, ANC, and platelet nadirs occurred at the treatment midpoint, with a similar magnitude of decline, and remained below baseline thereafter [5]. These results can be explained by the lifespan of each blood cell. In agreement with Casarett’s classification, hematopoietic stem cells are the most radiation sensitive of cells (class 1; vegetative intermitotic cells), while polymorphonuclear granulocytes and erythrocytes are the most radiation resistant of cells (class 5; fixed postmitotic cells) [6]. Therefore, the concentration of each blood cell type depends mainly on their lifespan. Red blood cells (RBC) have a lifespan of approximately 120 days, whereas neutrophils and platelets have lifespans of about 5.4 days and 9–12 days, respectively. Compared to neutrophils and platelets, RBC can live long enough for bone marrow at unirradiated areas to be able to compensate for decreased RBC production or for bone marrow at the irradiated area to regenerate itself, while the others cannot. This is why the Hb level was not associated with cumulative dose, whereas WBC count, ANC, and platelet count were negatively associated with the variable.

On the basis of dosimetric parameters, the Hb level was reported to be affected by a specific radiation dose delivered to a defined volume. In a systematic review by Corbeau et al. on the correlation between pelvic bone marrow radiation dose and hematologic toxicity in cervical cancer treated with CCRT, only V10 and V20 were found to be correlated with Hb nadirs [7]. Whole iliac bone V20 > 90% was revealed by Lewis et al. to be predictive of grade ≥ 2 anemia in cervical cancer patients receiving postoperative CCRT with IMRT [8]. Among patients with cervical cancer treated with postoperative CCRT using IMRT, whole-pelvis bone marrow (WP) V30, V40, V45, and V50; iliac bone marrow (IL) V20, V40, V45, and V50; and lower pelvic bone marrow (LP) V30, V40, V45, and V50 were associated with anemia in a correlation analysis presented by Yang et al. In multiple regression analysis, the side effect was negatively correlated with WP V30, IL V40, and LP V40 [9]. Regarding the data of cervical cancer patients treated with CCRT and IMRT published by Chen and colleagues, Hb nadirs were positively correlated with baseline values and negatively correlated with relative lower pelvis V10, V25, V50, and Dmean. The cutoff values of these variables were 96.150 g/L, 74.62%, 44.290%, 7.258%, and 2020.850 Gy, respectively [10]. These findings conflict with the results of the present study. All the mentioned studies demonstrate that a sufficient dose delivered to an adequate volume could lead to significant Hb level reduction. However, in the previously mentioned systematic review, dosimetric parameters including V10, V20, V30, and Dmean were correlated with WBC nadirs. The results were consistent with ANC nadirs. Additionally, the baseline values of each parameter were shown to be predictive for their nadir values. These relationships were comparable to the present study. Nevertheless, no dose–volume parameter was reported to influence the platelet count in the systematic review [7].

There was a significant imbalance between the development and validation cohorts due to the use of random sampling in the present study. However, this imbalance could help in examining the generalizability of the models to populations that differ substantially from the development cohort. To evaluate whether the model is valid, performance metrics should be considered. With acceptable values in both development and internal validation datasets, the next step should involve external validation. In the present study, it could be agreed that the performance metrics for Hb level were admissible, with fairly high adjusted R‑squared and low mean absolute error (MAE), mean squared error (MSE), and root mean squared error (RMSE). Compared to the other parameters with adjusted R‑squared less than 50%, as well as large MAE, MSE, and RMSE, caution should be exercised during interpretation of the results. To improve the performance of the models, other regression analyses should be used to augment R‑squared and minimize the errors. In addition, machine learning (ML) has been shown to precisely predict outcomes in both medical and nonmedical scenarios in a vast number of publications and media [11, 12]. Commencing ML for CBC parameter prediction could represent a huge step in the development of patient care for radiotherapy, accurately identifying patients who require CBC monitoring during radiation treatment, which could result in better quality of life for patients and avoid superfluous expense.

A more pronounced incidence of grade ≥ 3 neutropenia in the elderly (≥ 70 years) was reported in the secondary analysis of the RTOG 94–10 trial comparing CCRT with different fractionation schemes to sequential chemoradiotherapy in patients with non-small cell lung cancer (NSCLC) [13]. The result was consistent with the secondary analysis of the NCCTG 94–24–52 trial comparing different CCRT fractionations in NSCLC patients. The authors revealed that elderly patients (≥ 70 years) had more grade ≥ 4 hematologic toxicity and leukopenia than younger ones [14]. On the contrary, in a nomogram established from the data of 3786 patients receiving radiotherapy alone by Takeda and colleagues, for the population of age 70 years and older, the older the patient gets, the less neutropenia occurs; however, this association was not found for anemia [15]. The results of the previous studies were inconsistent with those of the present study. Only Hb level and platelet count were significantly negatively correlated with age, and the associations were very weak. Notably, there were only 66 courses with patients aged at least 70 years (13.3%) in the present study. Given the small elderly population, the results are unlikely to be comparable to those of other studies. Nevertheless, there was no association found between hematological complications and age in the systematic review by Corbeau et al. [7]. This emphasizes the need for further research to investigate the association between patient age and hematological toxicity with exceptionally robust methodology.

Due to being a retrospective study, missing data can be expected, and neither randomization nor blinding was performed. Hence, the results should be interpreted with caution. On top of that, since all the radiation treatments were conducted using 3DCRT, it is questionable whether the results can be generalized to IMRT, especially in terms of cumulative dose, because bone marrow tends to receive different doses between these techniques. Besides, external validation with different datasets is needed before implementation of the model to evaluate its reproducibility and particularly generalizability, making sure that the model is transportable to different populations.

In conclusion, CBC parameters during radiotherapy were positively correlated with their baseline values. For Hb level, the model could explain the variance fairly; nonetheless, it was not significantly associated with the received cumulative dose. Although external validation is essential before implementation of the model is merited, the results could be used as a guide to individualize patient care before and during radiation treatment, especially for specifying patients who might require special attention or benefit from prophylactic management.

## Supplementary Information

Supplementary tables
