# Supplementary material for: Linear regression analysis for complete blood count parameters during radiotherapy
Source: Strahlenther Onkol. 2025 Jan 10;201(5):561–6. doi: 10.1007/s00066-024-02344-1 (PMC12014696; doi:10.1007/s00066-024-02344-1)
Supplement: Supplementary file 1 — Supplementary tables [file 66_2024_2344_MOESM1_ESM.docx]

**Table S1. Pearson correlation between CBC parameters during radiation treatment and patient and treatment characteristics**

| **Outcomes (p-value)** | **Baseline value** | **Age** | **Female** | **Male** | **Cumulative dose** | **CCRT** | **Breast** | **Pelvis** | **Brain** | **Bone** | **Head & neck** | **Thorax** |
| --- | --- | --- | --- | --- | --- | --- | --- | --- | --- | --- | --- | --- |
| **Hb** | 0.78  (<0.001) | -0.15  (<0.001) | -0.09  (<0.001) | 0.09  (<0.001) | 0.005  (0.83) | -0.18  (<0.001) | 0.13  (<0.001) | -0.18  (<0.001) | 0.11  (<0.001) | 0.02  (0.5) | -0.09  (<0.001) | 0.02  (0.33) |
| **WBC** | 0.41  (<0.001) | 0.02  (0.37) | -0.14  (<0.001) | 0.14  (<0.001) | -0.09  (<0.001) | -0.04  (0.08) | -0.14  (<0.001) | -0.05  (0.04) | 0.22  (<0.001) | 0.08  (<0.001) | 0.01  (0.68) | 0.06  (0.01) |
| **Neutrophil** | 0.46  (<0.001) | -0.03  (0.21) | -0.1  (<0.001) | 0.1  (<0.001) | 0.2  (<0.001) | 0.28  (<0.001) | 0.31  (<0.001) | 0.25  (<0.001) | 0.07  (0.005) | 0.01  (0.58) | 0.05  (0.02) | 0.03  (0.24) |
| **ANC** | 0.38  (<0.001) | 0.03  (0.28) | -0.14  (<0.001) | 0.14  (<0.001) | -0.05  (0.03) | 0.002  (0.93) | -0.16  (<0.001) | -0.01  (0.7) | 0.19  (<0.001) | 0.09  (<0.001) | 0.02  (0.52) | 0.06  (0.01) |
| **Platelet** | 0.60  (<0.001) | -0.05  (0.03) | -0.12  (<0.001) | 0.12  (<0.001) | -0.2  (<0.001) | -0.1  (0.57) | -0.14  (<0.001) | -0.004  (0.87) | 0.07  (0.004) | 0.12  (<0.001) | 0.08  (<0.001) | 0.09  (<0.001) |

ANC; Absolute neutrophil count, CCRT; Concurrent chemoradiation, Hb; Hemoglobin, WBC; White blood cell

**Table S2. Performance metrics of linear regression analysis for each each CBC parameter during radiation treatment**

|  | **Data** | **MAE** | **MSE** | **RMSE** | **Adjusted R2** |
| --- | --- | --- | --- | --- | --- |
| **Hb** | **Development** | 0.69 | 0.93 | 0.96 | 0.6 |
|  | **Validation** | 0.16 | 0.17 | 0.42 | 0.71 |
| **WBC** | **Development** | 1620.78 | 28888053.81 | 5374.76 | 0.42 |
|  | **Validation** | 335.29 | 1112806.72 | 1054.9 | 0.11 |
| **Neutrophil** | **Development** | 7.04 | 85.6 | 9.14 | 0.36 |
|  | **Validation** | 1.8 | 20.05 | 4.48 | 0.32 |
| **ANC** | **Development** | 1515.6 | 25834661.47 | 5082.78 | 0.42 |
|  | **Validation** | 341.8 | 1138992.86 | 1067.24 | 0.06 |
| **Platelet** | **Development** | 49140.68 | 5100593352 | 71418.44 | 0.43 |
|  | **Validation** | 10320.89 | 776116768.1 | 27858.87 | 0.36 |

ANC; Absolute neutrophil count, Hb; Hemoglobin, MAE; Mean absolute error, MSE; Mean squared error, RMSE; Root mean squared error, WBC; White blood cell

**Table S3. Interception and coefficients for multiple linear regression analysis of each CBC parameter during radiation treatment**

|  |  |  | **Coefficients (95%CI)** | | | | | | | | | |
| --- | --- | --- | --- | --- | --- | --- | --- | --- | --- | --- | --- | --- |
| **Outcomes** | **Interception (95%CI)** | **p-value** | **Baseline value** | **p-value** | **Age** | **p-value** | **Male** | **p-value** | **Cumulative dose** | **p-value** | **CCRT** | **p-value** |
| **Hb** | 3.43 (3.01 to 3.85) | < 0.001 | 0.73 (0.7 to 0.76) | < 0.001 | -0.006 (-0.01 to -0.002) | 0.001 | 0.16 (0.04 to 0.29) | 0.01 | 0.001 (-0.002 to 0.004) | 0.38 | -0.18 (-0.33 to -0.03) | 0.02 |
| **WBC** | 1579.79 (308.9 to 2850.68) | 0.02 | 0.61 (0.54 to 0.68) | < 0.001 | 15.33 (-3.22 to 33.88) | 0.11 | 892.11 (270.27 to 1513.95) | < 0.001 | -31.43 (-47.48 to -15.37) | < 0.001 | -414.97 (-1207.89 to -377.96) | 0.31 |
| **Neutrophil** | 34.2 (30.73 to 37.66) | < 0.001 | 0.48 (0.44 to 0.52) | < 0.001 | -0.03 (-0.07 to 0.001) | 0.06 | -1.35 (-2.5 to -0.19) | 0.02 | 0.15 (0.12 to 0.18) | < 0.001 | 4.99 (3.51 to 6.46) | < 0.001 |
| **ANC** | 866.82 (-307.03 to 2040.67) | 0.15 | 0.59 (0.51 to 0.66) | < 0.001 | 14.25 (-3.34 to 31.84) | 0.11 | 656.99 (67.48 to 1246.5) | 0.03 | -15.03 (-30.25 to 0.19) | 0.05 | -45.51 (-797.53 to 706.5) | 0.91 |
| **Platelet** | 116654.7 (97219.7 to 136089.7) | < 0.001 | 0.53 (0.5 to 0.56) | < 0.001 | 116.89 (-144.01 to 377.79) | 0.38 | 5038.69 (-3662.67 to 13740.04) | 0.26 | -1232.76 (-1457.41 to -1008.12) | < 0.001 | -19246.88 (-30357.68 to -8136.07) | < 0.001 |

|  | **Coefficients (95%CI)** | | | | | | | | | |
| --- | --- | --- | --- | --- | --- | --- | --- | --- | --- | --- |
| **Outcomes** | **Pelvis** | **p-value** | **Brain** | **p-value** | **Bone** | **p-value** | **Head & neck** | **p-value** | **Thorax** | **p-value** |
| **Hb** | -0.18 (-0.33 to -0.03) | 0.02 | 0.22 (0.05 to 0.38) | 0.01 | -0.34 (-0.53 to -0.15) | < 0.001 | -0.94 (-1.26 to -0.62) | < 0.001 | -0.31 (-0.68 to 0.06) | 0.1 |
| **WBC** | -187.88 (-976.18 to 600.42) | 0.64 | 1867.69 (976.06 to 2759.31) | < 0.001 | 1500.05 (506.09 to 2494.02) | < 0.001 | -1568.48 (-3222.96 to 86.01) | 0.06 | 529.81 (-1412.08 to 2471.71) | 0.59 |
| **Neutrophil** | 4.44 (2.97 to 5.9) | < 0.001 | 3.97 (2.37 to 5.58) | < 0.001 | 3.5 (1.65 to 5.36) | < 0.001 | 4.09 (1.04 to 7.15) | 0.01 | 5.73 (2.14 to 9.31) | 0.002 |
| **ANC** | 192.12 (-554.67 to 938.92) | 0.61 | 1718.94 (867.82 to 2570.06) | < 0.001 | 1640.19 (696.23 to 2584.15) | < 0.001 | -1072.96 (-2646 to 500.07) | 0.18 | 942.47 (-902.82 to 2787.75) | 0.32 |
| **Platelet** | 11943.6 (929.1 to 22958.09) | 0.03 | 23189.59 (11265.68 to 35113.5) | < 0.001 | 35660.2 (21753.53 to 49566.88) | < 0.001 | 50432.33 (27493.52 to 73371.15) | < 0.001 | 55060.73 (28054.57 to 82066.89) | < 0.001 |

ANC; Absolute neutrophil count, CCRT; Concurrent chemoradiation, Hb; Hemoglobin, WBC; White blood cell
